# Identification and Characterization of “Candidatus Rickettsia Thierseensis”, a Novel Spotted Fever Group Rickettsia Species Detected in Austria

**DOI:** 10.3390/microorganisms8111670

**Published:** 2020-10-28

**Authors:** Anna-Margarita Schötta, Michiel Wijnveld, Dieter Höss, Gerold Stanek, Hannes Stockinger, Mateusz Markowicz

**Affiliations:** 1 Institute for Hygiene and Applied Immunology, Center for Pathophysiology, Infectiology and Immunology, Medical University of Vienna, Kinderspitalgasse 15, A-1090 Vienna, Austria; michiel.wijnveld@meduniwien.ac.at (M.W.); gerold.stanek@meduniwien.ac.at (G.S.); hannes.stockinger@meduniwien.ac.at (H.S.); mateusz.markowicz@meduniwien.ac.at (M.M.); 2 Private Medical Office, Vorderthiersee 19, 6335 Thiersee, Austria; hoess.thiersee@a1business.at

**Keywords:** Austria, spotted fever group Rickettsia, Ixodes ricinus, ticks, Candidatus Rickettsia thierseensis

## Abstract

*Rickettsia* spp. are the second most common pathogens detected in *Ixodes ricinus* ticks in Austria after *Borrelia burgdorferi* sensu lato. Species belonging to the spotted fever group (SFG) are the causative agents for tick-borne rickettsiosis across the world. So far, only four SFG *Rickettsia* spp. were detected in Austria, namely *R. helvetica*, *R. raoultii*, *R. monacensis* and *R. slovaca*. Here, we describe the identification of a new SFG *Rickettsia* species detected in an *I. ricinus* tick. Sequencing of various rickettsial genes revealed a nucleotide sequence similarity of 99.6%, 98.5%, 97.3% and 98.5% to the *gltA*, *ompA*, *ompB*, and *sca4* genes, respectively, of known and validated species. Additionally, sequencing of the *htrA* gene and 23S-5S intergenic spacer region also only showed 99.6% and 99.2%, respectively, similarity to known species. Therefore, and in accordance with current criteria for *Rickettsia* species discrimination, we hereby describe a new species of the SFG with putative pathogenic potential. We propose the name “*Candidatus* Rickettsia thierseensis” based on the village Thiersee in the Austrian province of Tyrol, where the carrying tick was found.

## 1. Introduction

*Rickettsia* spp. are obligate intracellular bacteria that can be divided into several groups: (a) the spotted fever group (SFG) is the largest one. It contains strains commonly found in ticks, mites and fleas that can cause spotted fever in humans. (b) The typhus group (TG) comprises *R. prowazekii* and *R. typhi*, which are transmitted by lice and fleas and cause typhus in humans. (c) The ancestral group is based on the most outlying *Rickettsia* spp. which are *R. canadensis* and *R. belli.* (d) Lastly, the so-called transitional group has recently been described which includes species previously assigned to the SFG, however, this group is still discussed controversially [1,2]. Some species that were previously not considered pathogenic to humans are nowadays proven pathogens [3]. Thus, surveillance studies investigating the presence of *Rickettsia* spp. circulating in our environment—especially within arthropods, like ticks that can act as vectors—are of great medical importance. 

In Austria, *Ixodes ricinus* (*I. ricinus*) is the most abundant vector of zoonotic diseases and, therefore, the most dangerous tick species from a medical point of view. The prevalence of *Rickettsia* spp. in the Austrian *I. ricinus* population ranges between 7% and 50% depending on the collection site [4]). Thus, besides *Borrelia burgdorferi* sensu lato, *Rickettsia* spp. of the SFG species are the most frequent pathogens detected in ticks. Until now, the detected SFG rickettsiae in Austria comprise the genospecies *R. helvetica*, *R. raoultii*, *R. monacensis* and *R. slovaca* [4]. 

Here, we describe a new SFG *Rickettsia* sp. which we found in a tick obtained from a healthy human host in Thiersee, a village in the Austrian province of Tyrol. We characterized this species using various genes commonly used for the discrimination of *Rickettsia* spp. in agreement with gene sequence-based criteria used in tick-borne *Rickettsia* research [5], as well as by performing a multigenic molecular approach. All assays performed confirm the presence of a new SFG *Rickettsia* sp.

## 2. Materials and Methods 

### 2.1. Tick Identification and DNA Extraction

The unengorged tick was obtained from a healthy person who participated in a study at our institute. The study was approved by the Ethical Committee of the Medical University of Vienna (1064/2015, approval date: 08/06/2015) and of the Medical University of Innsbruck (AN2016-0043-359/4.16, approval date: 09/05/2016). According to the study participant, the tick was removed from the right hand within the first hour of attachment.

Morphological identification was done using a stereomicroscope in combination with an *Ixodes* spp. identification key. Additionally, molecular identification was performed based on the mitochondrial 16S rRNA gene using primers 16S+1 (5′-CTGCTCAATGATTTTTTAAATTGCTGTGG-3′) and 16S-1 (5′-CCGGTCTGAACTAGATCAAGT-3′) [6]. 

DNA was extracted using the Qiagen DNeasy Blood & Tissue Kit (Qiagen, Hilden, Germany) as previously described [4]. 

### 2.2. PCRs of Rickettsial Genes

The following genes were investigated for the molecular characterization of the unknown SFG sp.: citrate synthase (*gltA*), outer membrane protein A (*ompA*) and B (*ompB*), cell surface antigen 4 (*sca4* or geneD), 17-kDa outer membrane antigen (*htrA*), 16S rDNA and the 23S-5S IGS. Table 1 gives an overview of the primers used. 

The PCRs were performed with the Phire Hot Start II polymerase kit (Thermo Fisher Scientific, Vienna, Austria) using 5 µL Phire reaction buffer, 400 nM dNTPs each (Solis Biodyne, Tartu, Estonia), 400 nM of each primer, 0.5 µL Phire II polymerase and 2.5 µL template DNA (1:5 diluted) and PCR-grade H2O (Sigma-Aldrich, Darmstadt, Germany) in a total volume of 25 µL. In the case of unsuccessful PCR of certain fragments, the mastermix was adjusted by using 200 nM dNTPs each and 0.125 µL Phire II polymerase that improved some PCRs. All PCRs were run in a Biorad Touch C-1000 thermal cycler (Hercules, CA, USA) using the following protocol: an initial denaturation step of 98 °C for 30 sec, followed by 45 cycles of 98 °C for 5 sec, respective annealing temperature (Ta) °C for 5 s and 72 °C for 15 s and, lastly, a final elongation step at 72 °C for 30 s.

Amplification of the *gltA* gene was performed using two PCRs spanning a total of 1150 bp. The first fragment (I) with a product length of 398 base pairs (bp) was amplified at 50 °C Ta, the second fragment (II) of 777 bp was run at 55 °C Ta. 

The *ompA* gene sequences were obtained by using five different PCRs; fragment I was the amplicon of the first part of the gene. The following tandem repeat region was not covered by the PCRs. Fragments II to V were assembled into a 3221 bp long consensus sequence. Fragment I (632 bp) was run at 55 °C Ta, fragment II (819 bp), IV (889 bp) and V (892 bp) at 50 °C Ta and fragment III (901 bp) at 46 °C Ta. 

The *ompB* gene sequence resulted from seven PCRs, all of which were run with a Ta of 50 °C. The product lengths were 856, 881, 1022, 876, 812, 885 and 648 bp, for fragments I to VII, respectively, resulting in a 4934 bp spanning region upon assembling. 

Amplification of *sca4* (gene D) consisted of five PCRs performed at 50 °C Ta. The fragment sizes were 910, 624, 661, 745 and 738 bp, for fragments I to V, respectively. Together they spanned a region of 3078 bp of the gene. 

The PCR of the 17kDa antigen (*htrA*) was run at 55 °C Ta, resulting in a 549 bp long fragment covering the whole gene.

The amplification of the 16S rDNA was performed with the following protocol: 98 °C for 30 s followed by 45 cycles at 98 °C for 10 s, 58 °C for 10 sec, 72 °C for 20 s and a final elongation for 30 s at 72 °C. The obtained PCR product was 1462 bp long.

Finally, the 23S-5S IGS PCR was run with a touchdown program, as described previously [4]. Depending on the species, the product length of this target varied between 341 and 413 bp (http://insilico.ehu.eus/PCR/). 

### 2.3. PCR Purification and Sequencing

The entire PCR reaction volume was loaded onto a 1% agarose gel containing GelRed (Biotrend, Cologne, Germany). The bands were excised and purified using the QIAquick Gel Extraction kit (Qiagen, Hilden, Germany). The purified amplicons were sent to Microsynth (Microsynth Austria GmbH, Vienna, Austria) for bidirectional sequencing. 

Certain PCR products were cloned into a plasmid for sequencing in order to gather full-length sequences of the gene endings. To this end, the purified fragments were ligated into the pJET1.2 blunt end cloning vector and cloning was performed with the CloneJET PCR cloning kit (Thermo Fisher Scientific, Vienna, Austria) using DH5α cells according to the manufacturer’s instructions. The plasmids were extracted using the GeneJet Plasmid Miniprep kit (Thermo Fisher Scientific, Vienna, Austria) and sent in for sequencing (Microsynth Austria GmbH, Vienna, Austria). 

The obtained sequenced fragments were assembled and analyzed using MEGA7 [13]. 

### 2.4. Data Availability

The sequences obtained during this study were submitted to GenBank and are available under the following accession numbers: MT424977-MT424983.

## 3. Results

We performed the phylogenetic analyses in MEGA7 [13] singularly for each gene using reference sequences of strains, for which whole-genome sequence data were available. Additionally, other strains (e.g., *Candidatus* Rickettsia spp. or species for which complete genome data were not yet available) were also included in the single-gene analyses in the case that the available sequences were long enough. 

In the single-gene analyses, all phylogenetic trees were constructed using the neighbor-joining algorithm with the Kimura 2-parameter method and a discrete Gamma distribution inferred from 1000 bootstrap replicates. Trees were drawn to scale with branch lengths indicating the evolutionary distance. Gaps and missing data were deleted. The accession numbers and strain designations of the genospecies included in the different analyses are displayed in the phylogenetic trees.

### 3.1. Phylogenetic Analysis of the gltA Gene

After cloning and assembling the two PCR fragments of *gltA* in MEGA7, a total consensus sequence of 1150 bp was obtained covering *gltA* gene positions 82 to 1231, when compared to reference strains of the SFG. An initial DNA comparison using the Basic Local Alignment Search Tool (BLAST) showed 99.57% similarity to *R. parkeri* strain Atlantic Rainforest (CP040325.1) as well as similarity to some *R. sibirica* strains (e.g., KU310587.1). 

**Figure 1:**
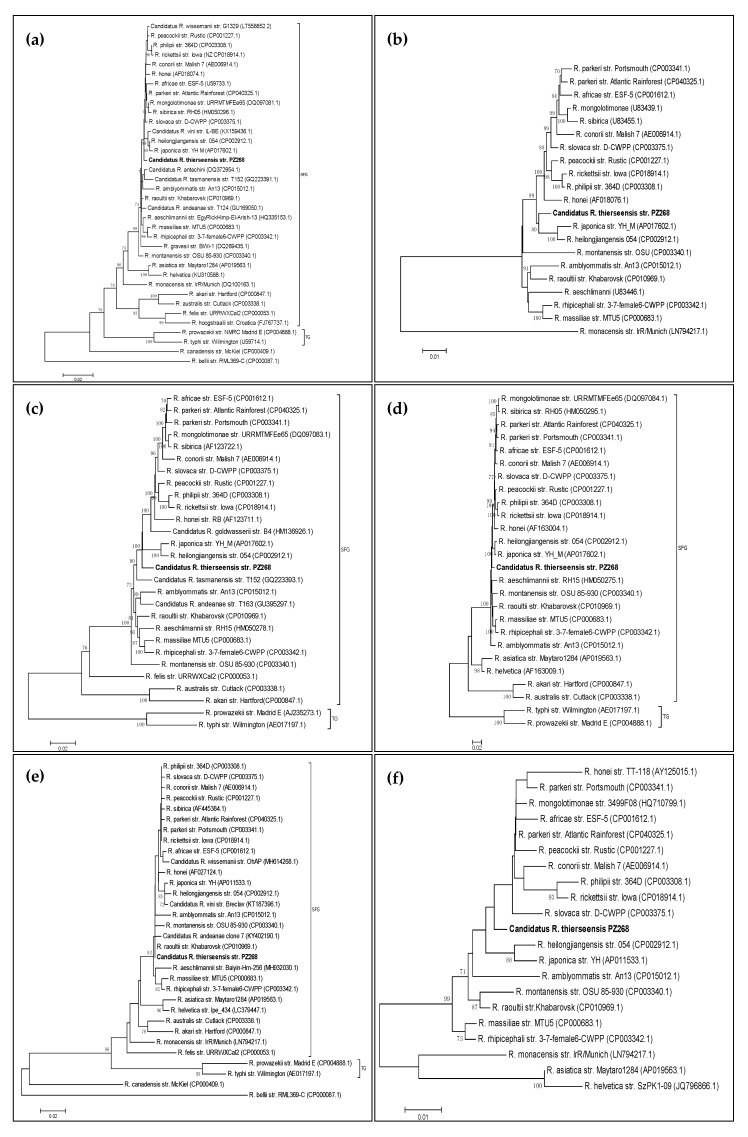
Phylogenetic trees for single genes. Neighbor-joining trees constructed with the Kimura 2-parameter method and inferred from 1000 bootstrap replicates. Only bootstrap values ≥70% are shown. The trees were drawn to scale with branch lengths indicating evolutionary distances. Gaps and missing data were deleted. Our strain under study is displayed in bold letters. (**a**) *gltA* gene showing 36 *Rickettsia* strains using a total of 1051 positions in the final dataset; (**b**) *ompA* gene including 21 strains and 3144 positions; (**c**) *ompB* gene including 28 strains and 4739 positions; (**d**) *sca4* gene displaying 26 strains and constructed from 2010 positions; (**e**) *htrA* gene showing 32 strains using 413 positions; (**f**) 23S-5S intergenic spacer region for 21 strains and built from 345 positions in the final dataset.

In order to construct a phylogenetic tree of the *gltA* gene (Figure 1a), a total amount of 1051 nucleotide positions were included comprising 36 different *Rickettsia* strains. The tree covered gene positions 105 to 1155 for SFG *Rickettsia* spp. (or, in the case of *R. belli*, 105 to 1158). 

### 3.2. Phylogenetic Analysis of the ompA Gene

Concerning the *ompA* gene, we performed the analysis in two steps as we did not want to include the tandem repeat separating the gene segments of interest in the analysis. The first segment (fragment I) comprised a sequence of 632 bp. A BLAST search of this fragment resulted in 96.20% identity to *R. raoultii* isolate Alear06 (KX506738.1). The second segment was obtained by assembling fragments II to V of the *ompA* PCRs, resulting in a 3221 bp long consensus sequence. The BLAST comparison showed 98.45% identity to various *R. heilongjiangensis* strains (e.g., AP019865.1) as well as to some *R. slovaca* strains (e.g., CP003375.1). 

The *ompA* gene is not available for all *Rickettsia* spp., especially not for most non-SFG *Rickettsia* spp. (e.g., *R. belli*, *R. typhi*, *R. prowazekii*). Therefore, they are not shown in the *ompA* tree. This also applies to *R. helvetica* and *R. asiatica* for which the *ompA* sequence is also not available even though they belong to the SFG. For fragment I, it was possible to include some transitional group *Rickettsia* spp., i.e., *R. akari*, *R. australis* and *R. canadensis*. However, for the longer fragment (II to V), these species also had to be excluded for an adequate alignment. The tree constructed for the longer fragment of the *ompA* gene containing 3144 nucleotide positions is shown in Figure 1b. 

### 3.3. Phylogenetic Analysis of the ompB Gene

After assembling the seven sequenced fragments of the *ompB* gene, we obtained a 4934 bp long spanning sequence, which displayed 97.34% identity to *R. slovaca* (CP003375.1 and CP002528.1). For the phylogenetic tree (Figure 1c), *R. belli* and *R. canadensis* had to be excluded to achieve an adequate alignment for analysis. Moreover, the SFG species *R. asiatica*, *R. monacensis* and *R. helvetica* were also excluded to construct the tree from a longer gene region spanning 4739 nucleotides.

### 3.4. Phylogenetic Analysis of the sca4 Gene (Gene D)

Upon assembling, the five PCR products of the *sca4* gene yielded a nucleotide sequence of 3078 bp. Just like for the *ompB* gene, the closest similarity was obtained for *R. slovaca* with 98.48% identity (CP003375.1 and CP002428.1). To construct the tree, *R. belli*, *R. felis*, *R. canadensis* and *R. monacensis* were excluded. The phylogenetic tree built from 2010 positions is shown in Figure 1d. 

### 3.5. Phylogenetic Analysis of the htrA Gene (17-kDa Antigen)

Amplification and cloning of the product of the *htrA* gene yielded a 547 bp long sequence that displayed the closest similarity (99.63% identity) to *R. rickettsii* (e.g., CP018914.1) and *R. philipii* (CP003308.1). The entire gene sequence of *htrA* comprising 480 nucleotides is contained within this assembly. When using the gene sequence without the flanking regions for database comparison, we found the closest similarity (99.79% identity) to *R. raoultii* strains (e.g., CP019435.1). The phylogenetic tree consisting of 32 sequences and covering gene positions 51 to 463 (413 nucleotide positions) is shown in Figure 1e.

### 3.6. Phylogenetic Analysis of the 16S rRNA Gene

The 1094 bp long consensus sequence obtained from the 16S rRNA gene yielded 100% identity to multiple *Rickettsia* strains belonging to the species *R. raoultii* (e.g., MK304546.1), *R. conorii* (e.g., MF002584.1) and *R. gravesii* (NR_157982.1). It is known that the 16S rRNA is not a valuable target for discriminating *Rickettsia* spp. and that many distinct species show 100% similarity to each other when comparing fragments of this gene. Therefore, due to low significance, we do not show a phylogenetic tree for this gene. 

### 3.7. Phylogenetic Analysis of the 23S-5S Intergenic Spacer (IGS)

We cloned and sequenced a 386 bp fragment of the 23S-5S IGS region and found the closest match (99.22% identity) to *R. parkeri* strain Atlantic Rainforest (CP040325.1). To enable enclosure of more positions (in total 345) in the analysis, we only used SFG *Rickettsia* spp. to draw the phylogenetic tree (Figure 1f). 

### 3.8. Multigenic Analysis of the Concatenated Sequences

**Figure 2:**
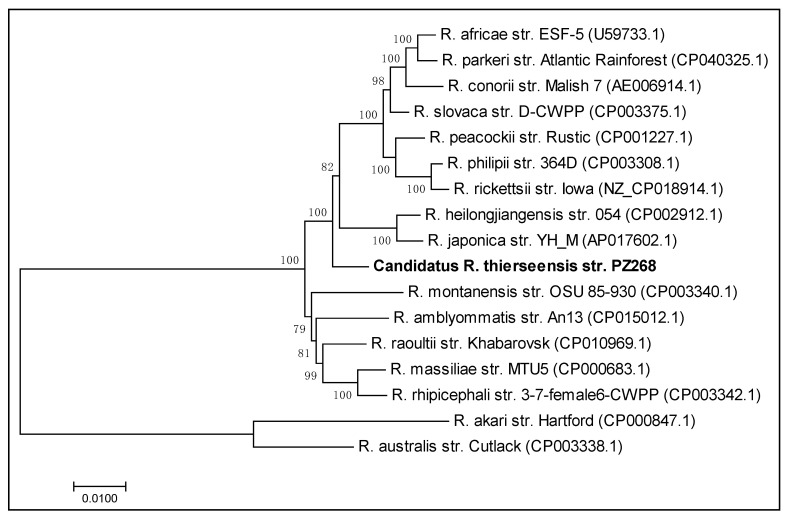
Maximum likelihood tree for multigenic phylogenetic analysis. Maximum likelihood tree built from 100 bootstrap replicates of the concatenated sequences of the *gltA*, *ompA*, *ompB*, *sca4* and *htrA* genes, respectively. The Tamura three-parameter model was applied using a discrete Gamma distribution. A total of 17 *Rickettsia* spp. were included and the final dataset contained 9920 positions. Our *Candidatus* Rickettsia thierseensis strain is indicated by bold letters.

To carry out a powerful multigene phylogenetic analysis, we selected 16 *Rickettsia* species and concatenated their *gltA*, *ompA*, *ompB*, *sca4* and *htrA* sequences. As a reference, we used *R. africae* strain ESF-5 (CP001612.1). Gaps and missing data were deleted, resulting in a different numbering of included nucleotides compared to the total spanning region. The *gltA* sequence covered gene positions 82 to 1231 with 1150 nucleotides included, the *ompA* sequence covered positions 1 to 587 with 574 nucleotides included, the *ompB* sequence covered positions 38 to 4965 with 4880 nucleotides included, the *sca4* sequence covered positions 1 to 3075 with 2840 nucleotides included and, finally, the *htrA* sequence covered gene positions 1 to 476 with all nucleotides included. Using these sequences, we constructed a maximum likelihood tree from 100 bootstrap replicates using the Tamura three-parameter model. To model evolutionary rate differences among sites, we applied a discrete Gamma distribution (+G, parameter = 0.5282). Finally, we constructed the tree from 9920 total nucleotide positions retrieved from the five rickettsial genes and the 17 different *Rickettsia* spp. including our *Candidatus* Rickettsia thierseensis sp. (Figure 2). The branch lengths indicate the number of substitutions per site. 

## 4. Discussion

We describe a new *Rickettsia* species of the SFG that we identified in an *I. ricinus* nymph, which bit a patient in Thiersee, a village in the Austrian province of Tyrol. We do not know during which stage of the tick’s life cycle this *Rickettsia* sp. was taken up or whether it was present within the tick as an endosymbiont and passed on transovarially. By negative serological testing of a paired sample, we at least ruled out the possibility that the tick had obtained this *Rickettsia* sp. from the human it was attached to. Moreover, the patient did not suffer from any symptoms evocative of rickettsiosis.

In our analysis, we clearly were able to allocate this new species to the SFG rickettsiae. There are several subgroups within the SFG, which are currently based around the species *R. tamurae*, *R. massiliae*, *R. japonica* and *R. rickettsii* [1]. In our multigenic analysis, our isolate branched between the *R. japonica* and *R. massiliae* groups. To characterize the novel *Rickettsia* sp., our aim was to cover the investigated genes as well as possible by using conventional molecular methods in an attempt to obtain long DNA sequences for analysis and facilitate submission to the GenBank database. Therefore, our data can easily be included in future phylogenetic comparisons of common rickettsial genes. 

The discovery of new and emerging tick-borne microorganisms has a considerable impact on future research into tick-borne diseases. A large number of tick-borne microorganisms were initially discovered in their tick host before infection in humans or animals was proven. For example, this was the case with *Candidatus* Neoehrlichia mikurensis, which represented the first case of neoehrlichiosis in an immunocompromised patient in 2009 [14], approximately 10 years after its discovery as an *Ehrlichia*-like organism in *I. ricinus* ticks in the Netherlands [15]. *Borrelia miyamotoi* was also first identified in a tick from Japan in 1995 [16] before being associated with human illness for the first time 17 years later [17].

Although *Rickettsia* spp. are among the oldest known zoonotic agents, the situation is similar [18]. Currently, the SFG *Rickettsia* spp. found in ticks from Austria comprise *R. helvetica*, *R. raoultii*, *R. monacensis* and *R. slovaca* [4]. The first detection of *R. helvetica* in humans was reported in 1999 [19], although it was already isolated from ticks in 1979 [20]. *R. raoultii* and its genetic variants have been known since 1999, yet it was discovered in the scalp of a patient with tick-borne lymphadenitis (TIBOLA) as late as 2002 [21]. *R. monacensis* was initially isolated and described in 2002 from ticks from Germany [22], and the first human case occurred in 2007 in Spain [23]. Finally, *R. slovaca*, which was first identified in 1968, was detected by molecular methods in a patient in 1996 [24]. These examples underscore the importance of studying, discovering and appropriately describing new tick-borne microorganisms, even though the pathogenic potential is not yet known. With respect to *Rickettsia* spp., the genus has undergone rapid expansion in the past few years, leading to a large number of newly described species of unknown pathogenicity. However, due to its intracellular lifestyle, cultivation of *Rickettsia* spp. is laborious and not easily possible, making it difficult to obtain cultured isolates. Therefore, new (*Candidatus*) *Rickettsia* spp. are mainly discovered via molecular methods, resulting in a high number of new strains and putative species that are not yet available in culture and, hence, leading to *Candidatus* status.

In accordance with the current criteria for defining a new species in the *Rickettsia* genus, a nucleotide sequence similarity of less than 99.9%, 99.3%, 98.8% and 99.2% to the *gltA*, *sca4*, *ompA* and *ompB* genes, respectively, is required [5]. Our isolate shows a similarity of 99.6%, 98.5%, 98.5% and 97.3% to the *gltA*, *sca4*, *ompA* and *ompB* genes, respectively, of known and validated species. Thus, the fulfillment of these selection criteria proves the presence of a new SFG Rickettsia species for which we propose the name *Candidatus* Rickettsia thierseensis.

## Figures and Tables

**Table 1:** Primers used for characterization of the novel *Rickettsia* sp.

Gene	Fragment	Primer Name	Primer Sequence (5′→3′)	Reference
*gltA*	I ^1^	CS-78	GCAAGTATCGGTGAGGATGTAAT	[7]
		CS-323	GCTTCCTTAAAATTCAATAAATCAGGAT	
	II ^1^	CS-239	GCTCTTCTCATCCTATGGCTATTAT	
		CS-1069	CAGGGTCTTCGTGCATTTCTT	
*ompA*	I ^1^	190-70	ATGGCGAATATTTCTCCAAAA	[8]
		190-701	GTTCCGTTAATGGCAGCATCT	
	II ^1^	190-3588	AACAGTGAATGTAGGAGCAG	
		190-4406	ACTATACCCTCATCGTCATT	
	III	190-4338	TTCAGGAAACGACCGTACG	
		190-5238	ACTATTAAAGGCTAGGCTATT	
	IV	190-5125	GCGGTTACTTTAGCCAAAGG	
		190-6013	TCTTCTGCGTTGCATTACCG	
	V ^1^	190-5917	TCAGGGAATAAAGGTCCTG	
		190-6808	CACGAACTTTCACACTACC	
*ompB*	I ^1^	M59 F	CCGCAGGGTTGGTAACTGC	[9]
		120-807 R	CCTTTTAGATTACCGCCTAA	
	II	120-607 F	AATATCGGTGACGGTCAAGG	
		120-1497	CCTATATCGCCGGTAATT	
	III	120-1378	CCTATATCGCCGGTAATT	
		120-2399	CTTGTTTGTTTAATGTTACGGT	
	IV	120-2113	CGATGCTAACGTAGGTTCTT	
		120-2988	CCGGCTATACCGCCTGTAGT	
	V	120-2788	AAACAATAATCAAGGTACTGT	
		120-3599	TACTTCCGGTTACAGCAAAGT	
	VI	120-3462	CCACAGGAACTACAACCATT	
		120-4346	CGAAGAAGTAACGCTGACTT	
	VII ^1^	120-4232	GGTTTCTCATTCTCTCTATATGG	
		120-4879	TTAGAAGTTTACACGGACTTTT	
*sca4*	I ^1^	D1f	ATGAGTAAAGACGGTAACCT	[10]
		D928r	AAGCTATTGCGTCATCTCCG	
	II	D767f	CGATGGTAGCATTAAAAGCT	
		D1390r	CTTGCTTTTCAGCAATATCAC	
	III	D1219f	CCAAATCTTCTTAATACAGC	
		D1876r	TAGTTTGTTCTGCCATAATC	
	IV	D1738f	GTATCTGAATTAAGCAATGCG	
		D2482r	CTATAACAGGATTAACAGCG	
	V ^1^	D2338f	GATGCAGCGAGTGAGGCAGC	
		D3069r	TCAGCGTTGTGGAGGGGAAG	
*htrA*	I ^1^	17k-5	GCTTTACAAAATTCTAAAAACCATATA	[7]
		17k-3	TGTCTATCAATTCACAACTTGCC	
16S rDNA	I	fD1	AGAGTTTGATCCTGGCTCAG	[11]
		rP1	ACGGTTACCTTGTTACGACTT	
23S-5S IGS	I ^1^	RCK/23-5-F	GATAGGTCRGRTGTGGAAGCAC	[12]
		RCK/23-5-R	TCGGGAYGGGATCGTGTGTTTC	

^1^ The fragments were cloned to obtain longer gene sequences.
